# Long-term outcomes following 90Y Radioembolization of neuroendocrine liver metastases: evaluation of the radiation-emitting SIR-spheres in non-resectable liver tumor (RESiN) registry

**DOI:** 10.1186/s12885-022-09302-z

**Published:** 2022-03-01

**Authors:** Thomas Y. Wong, Kevin S. Zhang, Ripal T. Gandhi, Zachary S. Collins, Ryan O’Hara, Eric A. Wang, Kirubahara Vaheesan, Lea Matsuoka, Daniel Y. Sze, Andrew S. Kennedy, Daniel B. Brown

**Affiliations:** 1 Department of Radiology and Radiological Sciences, Vanderbilt University Medical Center, 1161 21st Avenue S, CCC-1118 Medical Center North, Nashville, TN 37232 USA; 2 Department of Biostatistics, Vanderbilt University Medical Center, Nashville, TN USA; 3 Miami Cardiac and Vascular Institute, Baptist Hospital, Miami, FL USA; 4 Interventional Radiology, University of Kansas Medical Center, Kansas City, KS USA; 5 Interventional Radiology, University of Utah Medical Center, Salt Lake City, UT USA; 6 Interventional Radiology, Carolinas Medical Center, Charlotte, NC USA; 7 Interventional Radiology, Saint Louis University Hospital, St. Louis, MO USA; 8 Transplant Surgery, Vanderbilt University Medical Center, Nashville, TN USA; 9 Interventional Radiology, Stanford University Medical Center, Palo Alto, CA USA; 10 Radiation Oncology, Sarah Cannon Research Institute, Nashville, TN USA

**Keywords:** Neuroendocrine tumor, Metastases, Liver cancer

## Abstract

**Background:**

The goal of this study was to evaluate efficacy and safety of 90Y radioembolization for neuroendocrine liver metastases (NELM) in a multicenter registry.

**Methods:**

One hundred-seventy patients with NELM were enrolled in the registry (NCT 02685631). Prior treatments included hepatic resection (*n* = 23, 14%), arterial therapy (*n* = 62, 36%), octreotide (*n* = 119, 83%), cytotoxic chemotherapy (*n* = 58, 41%), biologic therapy (*n* = 49, 33%) and immunotherapy (*n* = 10, 6%). Seventy-seven (45%) patients had extrahepatic disease. Seventy-eight (48%), 61 (37%), and 25 (15%) patients were Eastern Cooperative Oncology Group (ECOG) performance status of 0, 1, or ≥ 2. Tumor grade was known in 81 (48%) patients: 57 (70%) were well-, 12 (15%) moderate-, and 12 (15%) poorly-differentiated. Kaplan-Meier analysis and log rank tests were performed to compare overall and progression-free survival (OS/PFS) by tumor location and grade. Toxicities were reported using Common Terminology Criteria for Adverse Events v.5. Cox Proportional Hazards were calculated for pancreatic primary, performance status, extrahepatic disease at treatment, unilobar treatment, baseline ascites, and > 25% tumor burden.

**Results:**

One, 2, and 3-year OS rates were 75, 62 and 46%, respectively. Median OS was 33 months [(95% CI: 25-not reached (NR)]. The longest median OS was in patients with pancreatic (42 months, 95% CI: 33-NR) and hindgut 41 months, 95% CI: 12-NR) primaries. The shortest OS was in foregut primaries (26 months; 95% CI: 23-NR; X^2^ = 7, *p* = 0.1). Median OS of well-differentiated tumors was 36 months (95% CI: 10-NR), compared to 44 (95% CI: 7-NR) and 25 (95% CI: 3-NR) months for moderate and poorly differentiated tumors. Median progression-free survival (PFS) was 25 months with 1, 2, and 3-year PFS rates of 70, 54, and 35%, respectively. Thirteen patients (7.6%) developed grade 3 hepatic toxicity, most commonly new ascites (*n* = 8, 5%) at a median of 5.5 months. Performance status of ≥2 (HR 2.7, *p* = 0.01) and baseline ascites (HR 2.8, *P* = 0.049) predicted shorter OS.

**Discussion:**

In a population with a high incidence of extrahepatic disease, 90Y was effective and safe in treatment of NELM, with median OS of 41 months for well differentiated tumors. Grade 3 or greater hepatic toxicity was developed in 7.6% of patients.

**Trial registration:**

NCT 02685631.

## Introduction

The incidence of neuroendocrine tumors (NET) is increasing from an annual incidence of 1.1/100,000 people in 1973 to 7.0 per 100,000 in 2012 [1]. Approximately one-quarter of neuroendocrine patients have metastatic disease at presentation and 80% eventually develop liver metastases [1, 2]. Development of hepatic metastases is associated with shorter 5- and 10-year overall survival (OS) [3]. Many patients are initially treated with somatostatin analogs, agents which limit hormone production and are also cytostatic [4–7]. Patients with paraneoplastic symptoms or progressive disease breaking through somatostatin analogs require additional therapy [7]. Recommendations for patients with progressive low- or intermediate-grade liver-dominant disease include everolimus, peptide-receptor radiation therapy (PRRT), and arterial therapy including bland embolization, chemoembolization, and radioembolization.

Selection of the therapeutic arterial modality for neuroendocrine liver metastases (NELM) varies widely without standardization. Multiple studies failed to identify a superior OS across different arterial techniques [8–11]. One retrospective study described longer OS with chemoembolization compared to TARE [12]. Similarly, periprocedural toxicity profiles from TARE were similar to other arterial therapies in several studies [3, 9, 10, 13, 14]. NET patients have projected OS of 27–35 months following TARE despite presenting with bilobar hepatic metastases [9, 14–16]. Recent literature described chronic imaging changes of portal hypertension in NET patients who had prolonged survival following radioembolization [17, 18]. Additionally, a recent report described a 13% incidence of chronic hepatic toxicity following TARE in NET patients [19]. All the studies above had 64 or fewer patients undergoing TARE. Given the expected multiyear survival, particularly with low-grade NELM, further definition of survival and toxicity from TARE to treat NELM would be helpful. Given the increasing utilization of PRRT, toxicity in patients who undergo both PRRT and TARE is an area of interest [20].

The Radiation-Emitting SIR-Spheres in Non-Resectable liver tumor (RESiN) registry (NCT 02685631) is a multicenter, prospective observational data collection tracking demographics, dosimetry, treatment response, and therapy toxicity of resin embedded Yttrium-90 microspheres (Sir-Spheres; Sirtex Medical, Woburn Massachusetts) in different tumor types. The purpose of the current manuscript is to further characterize efficacy and toxicity of TARE in patients with NELM.

## Materials and methods

A total of 170 patients (74 women/96 men) with NELM were enrolled in RESiN across 36 institutions between 2015 and 2020. Institutional review board approval was obtained at each site and all patients signed informed consent. Patients received TARE in interventional radiology at the participating centers as part of multidisciplinary decision making for their care. Inclusion criteria for RESiN included appropriateness for arterial therapy in a patient ≥18 years of age and ability of the patient to provide informed consent. Data was entered into a Research Electronic Data CAPture (REDCap) database. As RESiN is an observational registry, patients were treated and followed using local institutional guidelines. No incentives for compliance were provided to enrollees.

Table 1 outlines demographic information within the registry. The majority of patients were male (*n* = 96, 56%) and white (*n* = 140, 82%). Eastern Cooperative Oncology Group (ECOG) performance status scores were 1 or greater in greater than half the patients (*n* = 86, 51%). Of the 81 (48% of the registry) patients with available tumor grade using mitotic index: 57 (70%) were well-, 12 (15%) were moderate-, and 12 (15%) were poorly-differentiated. The most common primary site was midgut (*n* = 54, 36%) followed by foregut (*n* = 39, 26%), pancreatic (*n* = 36, 24%), and hindgut (*n* = 10, 7%). Thirteen patients (9%) had an unknown primary site. Fifteen patients (9% of the registry) had prior biliary interventions with Whipple procedure as the most common (n = 5, 31%). Each site followed its own protocol to prevent cholangitis or abscess from colonized bile ducts.

**Table 1:** Baseline demographics of the treatment group

Gender (*n* = 170)	Female	74 (44%)
	Male	96 (56%)
Age (Median [IQR])		65.5 [56.0–73.0]
Race (*n* = 170)	American Indian or Alaska Native	0 (0%)
	Asian	3 (2%)
	Black or African American	16 (9%)
	Native Hawaiian or Pacific Islander	1 (1%)
	White or Caucasian	140 (82%)
	Unknown	7 (4%)
	Other	3 (2%)
Ethnicity (*n* = 170)	Hispanic or Latino	19 (11%)
	Non-Hispanic	137 (81%)
	Unknown	14 (8%)
	Other	0 (0%)
Enrollment Year (*n* = 170)	2015	7 (4%)
	2016	37 (22%)
	2017	68 (40%)
	2018	26 (15%)
	2019	23 (14%)
	2020	9 (5%)
ECOG (*n* = 164)	0	78 (48%)
	1	61 (37%)
	2 or more	25 (15%)
Grade (*n* = 81)	Well Differentiated	57 (70%)
	Moderately Differentiated	12 (15%)
	Poorly Differentiated	12 (15%)
Tumor Site (*n* = 152)	Foregut	39 (26%)
	Midgut	54 (36%)
	Pancreas	36 (24%)
	Hindgut	10 (7%)
	Unknown	13 (9%)
Tumor Burden % (Median [IQR])		25.9 [11.9–49.8]
Tumor Location (*n* = 165)	Bilobar	77 (47%)
	Unilobar	88 (53%)
Extrahepatic Metastasis (*n* = 161)		
Yes (*n* = 77, 48%)	Lung	22 (29%)
	Lymph Nodes	22 (29%)
	Bone	14 (18%)
	Peritoneum	9 (12%)
	Small Bowel	6 (8%)
	Brain	1 (1%)
	Gastric	1 (1%)
	Large Bowel	1 (1%)
	Prostate	1 (1%)
	Uterus	1 (1%)
	Other	28 (36%)
No (*n* = 85, 52%)		
Hepatic Resection	Yes	23 (14%)
Systemic Therapy (*n* = 144)	Octreotide	119 (83%)
	Biologic	49 (34%)
	Cytotoxic	58 (40%)
Arterial Embolization (*n* = 166)	Yes	62 (37%)
	No	104 (63%)
Biliary Intervention (*n* = 15)	Metallic Stent	2 (13%)
	Plastic Stent	1 (7%)
	Percutaneous Biliary Drainage	1 (7%)
	Surgical Anastomosis	5 (31%)
	Other	7 (47%)
Ascites		10 (6%)
Baseline Laboratories	Bilirubin in mg/dL (Median [IQR])	0.9 [0.6–1.4]
	Albumin in g/dL (Median [IQR])	4.1 [3.8–4.3]
	ALT in u/L (Median [IQR])	42.0 [23.8–73.0]
	AST in u/L (Median [IQR])	52.0 [28.0–72.0]
	INR Ratio (Median [IQR])	1.1 [1.0–1.3]
	Creatinine in mg/dL (Median [IQR])	1.1 [0.9–1.4]
	Chromogranin A in ng/mL (Median [IQR])	543 [215–2981]
	Platelet Count in thousands/uL (Median [IQR])	239.5 [170.5–315.8]

*IQR* interquartile range

Median hepatic tumor burden at treatment was 26% (IQR: 11.8–49.7%). Seventy-seven patients (45%) had extrahepatic metastatic disease and ten patients (6%) had ascites. Before TARE, 23 patients underwent hepatic resection and 62 received arterial therapy. One hundred forty-four patients (85%) received cytostatic or systemic therapy. The most commonly prescribed agent was one of the octreotide analogs (*n* = 119/144, 83%). Fifty-eight patients (40%) underwent cytotoxic chemotherapy, 49 (34%) received targeted therapy, and 10 (6%) received immunotherapy as outlined in Table 2.

**Table 2:** Previous treatments received by patients in the study

Agent Type	Number Treated
**Alkylating Agent**	
Temozolomide	20
Cisplatin	8
Oxaliplatin	6
**Topoisomerase Inhibitor**	
Irinotecan	9
**Antimetabolite**	
Capecitabine	24
**DNA Synthesis Inhibitor**	
Etoposide	12
**m-TOR Inhibitor**	
Everolimus	42
Sirolimus	1
**VEGF Inhibitor**	
Sunitinib	6
**PD-1 Inhibitor**	
Pembrolizumab	4
Nivolumab	4
**CTLA-4 Blockade**	
Ipilimumab	2

Dosimetry methods were available in 94 patients (55%). Body surface area (BSA) was the most commonly utilized method (*n* = 86, 91%). One hundred and sixty-six patients (98%) underwent a single cycle of therapy, while four patients received more than one cycle of treatment as described in Table 3.

**Table 3:** Treatment history of patients undergoing more than one cycle of radioembolization

Patient Number	Area Treated	Treatment Date
1	Bilobar	1/24/2017
	Left Lobe	2/22/2017
	Bilobar	4/20/2018
	Right Lobe	8/7/2018
2	Bilobar	3/18/2018
	Left Lobe	2/20/2020
	Right Lobe	4/3/2020
3	Right Lobe	8/3/2016
	Bilobar	8/6/2019
	Bilobar	9/3/2019
4	Bilobar	1/3/2019
	Bilobar	2/11/2019
	Right Lobe	4/23/2019

In total, 82 patients (48%) underwent bilobar and 88 (52%) had unilobar treatment. Median prescribed activity was 1.3 GBq (IQR: 0.9–1.5 GBq) and 1.9 GBq (IQR: 1.7–2.2 GBq) for uni- and bilobar treatments, respectively. Use of peptide receptor radiotherapy (PRRT) before or after TARE was tracked.

Follow-up imaging and lab studies were obtained per operator and institutional protocols. Tumor response including progression was assessed utilizing Response Evaluation Criteria in Solid Tumors (RECIST 1.1) at 6 months after treatment given the reported median time to response of 4.9 months [21]. Objective response rate (ORR) was the sum of complete and partial responses. Disease control rate (DCR) was ORR plus stable disease. Patients were censored at the time of last contact and follow-up continued through August, 2021.

Kaplan-Meier analysis and log rank tests were performed to compare OS and PFS for the entire cohort as well as by tumor grade and location. Based on trends towards lower survival with pancreatic primaries in a meta-analysis, OS and PFS were also calculated for pancreatic primaries versus the remaining group [16]. PFS end points included: progressive disease at imaging, death without progression, or transition to hospice.

Toxicities were reported using Common Terminology Criteria for Adverse Events v.5. Any patients receiving PRRT were tracked for hepatic function toxicity. Additionally, the cohort was divided into two groups: enrollment prior to (2015–2017) and following (2018–2020) the publication of the NETTER-1 trial [22] to estimate changes in enrollment rate using an exact binomial test.

Cox proportional hazards regression was performed for the following factors at treatment: pancreatic primary tumor, ECOG score of 1 or ≥ 2, unilobar treatment, extrahepatic disease, ascites, and tumor burden of ≥25%. All values were significant at a *p* < 0.05. All statistics were calculated using R Foundation for Statistical Computing,version 4.1.1 (Vienna, Austria).

## Results

### Survival

**Fig. 1:**
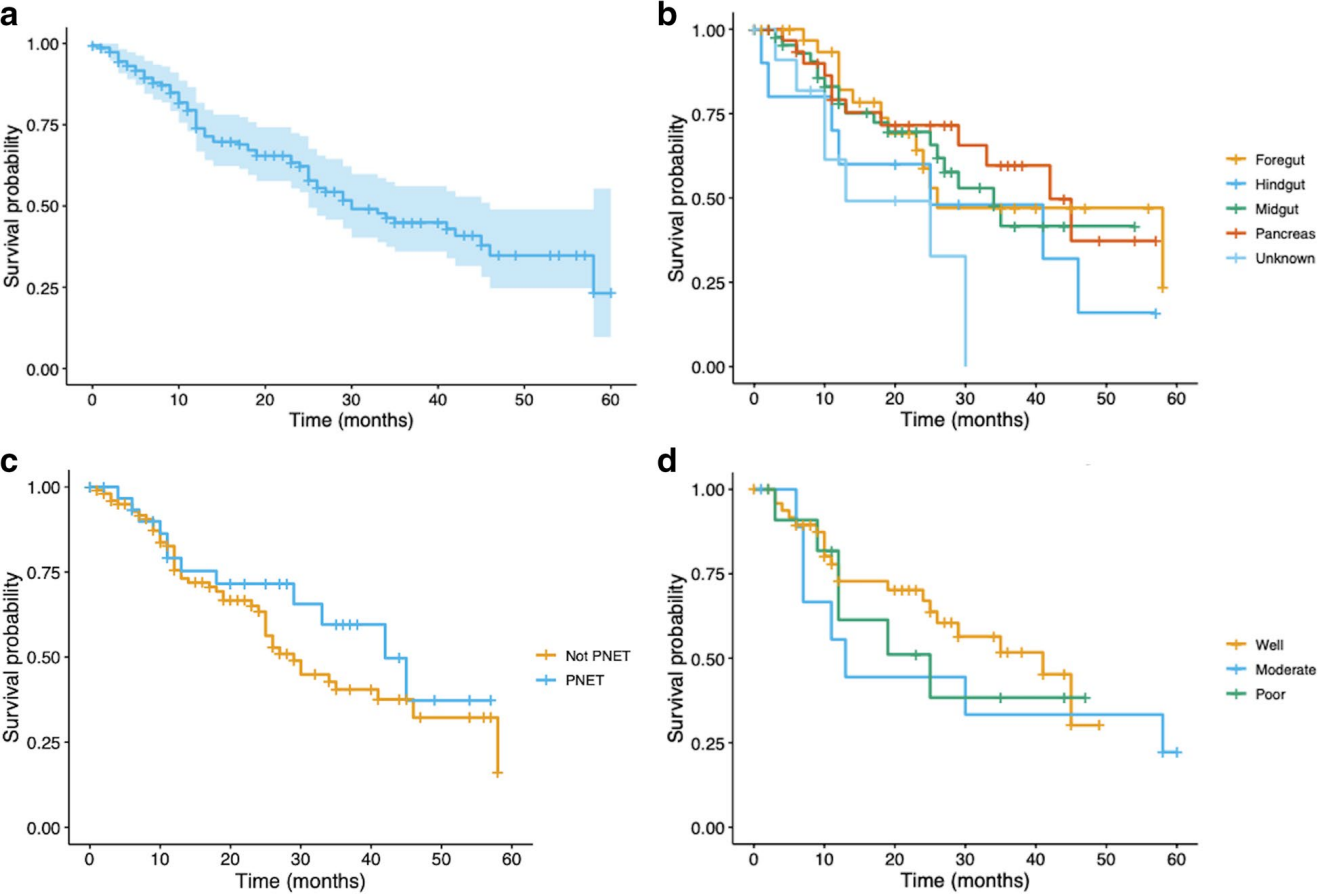
**A**-**D** Overall survival (**A**) for the entire cohort (**B**) by primary NET location (**C**) for Pancreatic primary (PNET) compared to all other primary tumors and (**D**) by tumor grade

Median OS for the entire cohort was 33 months [(95% CI: 25-not reached (NR)]. with 1-, 2-, and 3-year OS rates of 75, 62, and 46% (Fig. 1a). The differences in OS by primary tumor location were not statistically significant (Fig. 1b, X^2^ = 7.0, *p* = 0.1). The longest OS was in patients with pancreas (42 months; 95% CI: 33-NR) and hindgut tumors (41 months; 95% CI: 12-NR). OS was relatively shorter in midgut (35 months; 95% CI: 25-NR), foregut (26 months; 95% CI: 23-NR) and unknown primary locations (25 months; 95% CI: 10-NR). Pancreatic primary tumor OS (42 months; 95% CI: 33-NR) was longer than all other groups combined (29 months, 95% CI: 25-NR) although this outcome was not statistically significant (*p* = 0.3) (Fig. 1c). Well-differentiated tumors had a median OS of 41 months (95% CI: 26-NR), compared to 13 (95% CI: 7-NR) and 25 months (95% CI: 12-NR) for moderate- and poorly-differentiated tumors, respectively (Fig. 1d). This difference was not statistically significant (*p* = 0.67).

**Fig. 2:**
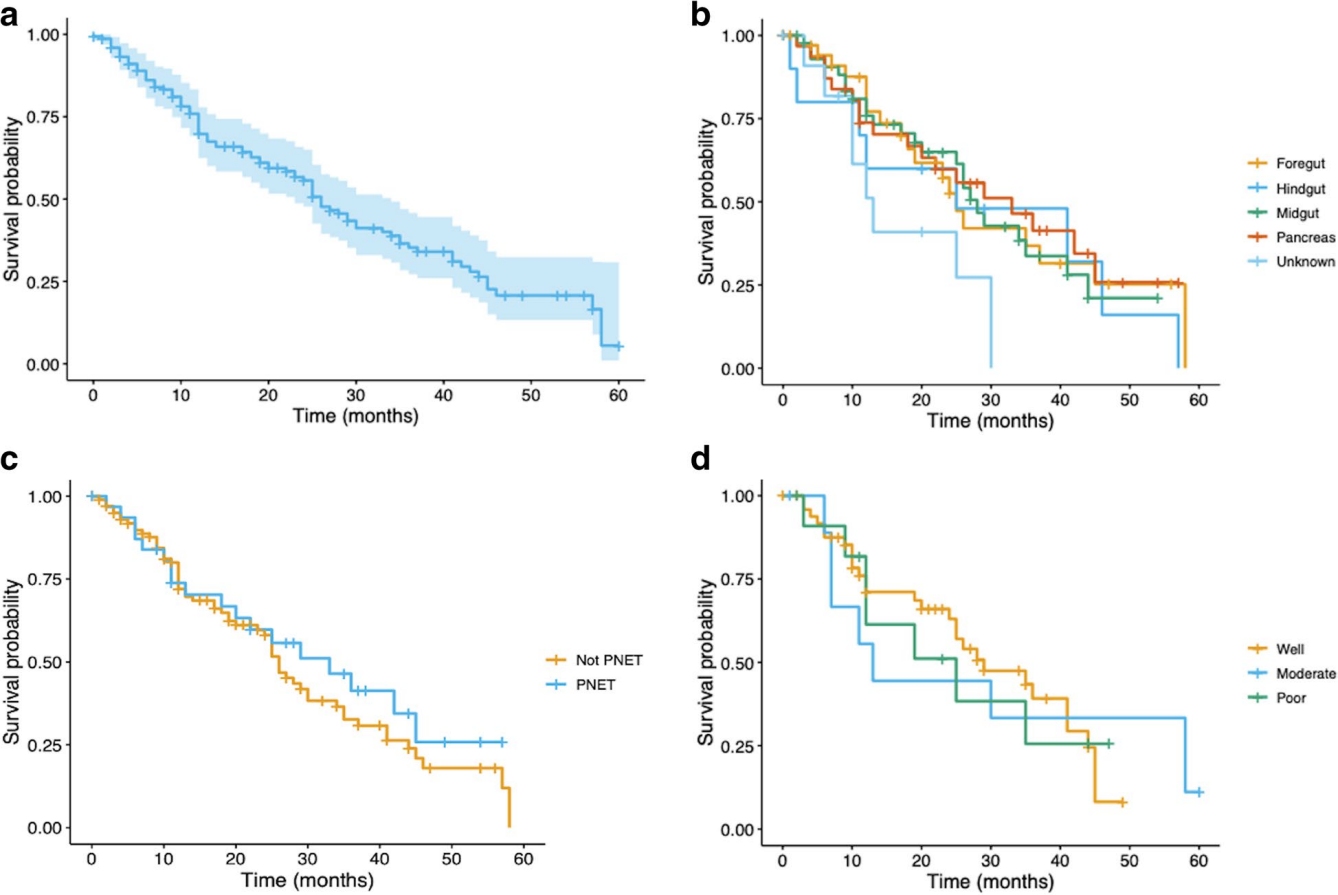
**A**-**D** Progression-Free Survival (**A**) for the entire cohort (**B**) by primary location (**C**) for pancreatic primary tumors (PNET) vs all other types combined and (**D**) by tumor grade

PFS for the entire group was 25 months (95% CI: 22–35 months) with 1-, 2-, and 3-year PFS rates of 70, 54, and 35% (Fig. 2a). There was no significant difference for PFS among primary locations (Fig. 2b,X^2^ = 4.8, *p* = 0.3). PFS was 2 years or greater for pancreatic (33 months, 95% CI: 18-NR), midgut (29 months, 95% CI: 20-NR), hindgut (25 months, 95% CI: 12-NR), and foregut (24 months, 95% CI: 18-NR) primaries, while 13 months (95% CI: 10-NR) for unknown primary location. Median PFS for pancreatic primary tumors (33 months, 95% CI: 20-NR) was longer than for all other tumor groups combined (26 months, 95% CI: 24–34; *p* = 0.4) (Fig. 2c). PFS was longest in well-differentiated tumors at a median of 35 months (95% CI: 25-NR), compared with 13 (95% CI: 7-NR) and 25 (95% CI: 12-NR) months in moderate- and poorly-differentiated tumors, respectively (Fig. 2d). This difference was not statistically significant (*p* = 0.96).

### Imaging response, progression and death

Post-treatment imaging was performed at 6 months on 121 patients. Ninety-nine patients had RECIST scoring performed. In this group, five (4%) had complete response, 39 (32%) had a partial response, 39 (32%) had stable disease and 16 (13%) had progressive disease. Forty-four patients (36%) had an objective response and 83 patients (69%) had disease control at 6 months. Seventy-one patients (42%) developed intrahepatic progressive disease. Twenty-six patients (37% of the cohort) developed progressive disease within a previously treated region while the remaining 45 patients (63%) with intrahepatic progression developed in an untreated area of liver. Sixty-one patients (36%) developed new extrahepatic disease. The most common site of new extrahepatic disease was skeletal (*n* = 23, 38%).

Sixty-seven patients (39% of the cohort) died, with data regarding cause of death available in 44 (66% of expired patients). Tumor progression was the most commonly described cause of death (*n* = 33, 49% of expired patients). One patient (2% of expired patients, 0.6% of the entire cohort) died of liver failure 11 months after treatment. Prior to TARE, this patient had received multiple types of chemotherapy including cisplatin and irinotecan. The remaining patients died of other causes. An additional 10 patients (6%) were lost to follow-up, and 6 (4%) signed into hospice. One patient left the study to seek treatment at a different institution and another left the study for unknown reasons.

### Cox proportional-Hazard model

The Cox Models for OS and PFS are in Table 5. Hazard ratios for OS were significant in patients with an ECOG score of 2 or greater (HR: 2.7, *p* = 0.01) and in the setting of baseline ascites (HR: 2.8, *p* = 0.049).

Similarly, hazard ratios for PFS were greater with an ECOG score of 2 or greater (HR: 2.4, *p* = 0.01) and baseline ascites (HR: 4.9, *p* = 0.0001). Patients with unilobar treatment (HR: 0.6, *p* = 0.03) and those with 25% or greater tumor burden (HR: 0.6, *p* = 0.049) also had a longer progression-free survival.

### Toxicities

Grade 3 or greater toxicities developed in 58 patients (34%) and are outlined in Table 4. Constitutional significant adverse events with an incidence of 5 (3%) or greater included: abdominal pain (*n* = 7, 4%) and anorexia (*n* = 5, 3%). Of the 15 patients with colonized bile ducts, one patient (7%) developed a hepatic abscess. Nineteen toxicities (20%) were attributed as definitely or probably related to treatment. These included all liver function toxicities in the absence of progressive hepatic disease including bilirubin (n = 5, 3%), new ascites (*n* = 8, 5%), alanine aminotransferase (*n* = 1, 0.6%) and alkaline phosphatase (n = 1, 0.6%). Of the 5 patients with Grade 3 hyperbilirubinemia, 2 normalized within 3 months, leaving 13 (7.6%) with durable hepatic function toxicities. The other three cases developed at 2.6, 8.9, and 11.5 months after TARE. Development of new ascites was identified a median of 5.5 months after treatment (range 3.5–18 months). Additional events included the death noted above (grade 5), two cases of abdominal pain (grade 3 and 4), one hepatic abscess (grade 3), and a single grade 3 lymphopenia.

**Table 4:** Toxicities and attributions

Adverse Events	Grade 3	Grade 4	Grade 5	Total
Abdominal Infection	1	0	0	1
Abdominal Pain	5	1	1	7
Acute Kidney Injury	1	0	0	1
Alanine Aminotransferase	1	0	0	1
Alkaline Phosphatase Increase	1	0	0	1
Anemia	2	0	0	2
Anorexia	5	0	0	5
Ascites, New	8	0	0	8
Bilirubin	5	0	0	5
Aspiration	1	0	0	1
Atrial Fibrillation	1	0	1	2
Chronic Kidney Injury	1	0	0	1
Death Not Otherwise Specified	0	0	24	24
Dehydration	3	0	0	3
Diarrhea	2	0	0	2
Duodenal Hemorrhage	1	0	0	1
Dyspnea	1	0	0	1
Edema, Cerebral	0	1	0	1
Edema, Limb	0	1	0	1
Encephalopathy	1	0	0	1
Fall	1	0	0	1
Febrile Neutropenia	2	0	0	2
Generalized Muscle Weakness	4	0	0	4
Headache	1	0	0	1
Hemorrhoids	1	0	0	1
Hepatic Failure	0	0	1	1
Hepatic Infection	2	0	0	2
Hyperglycemia	1	0	0	1
Hyperkalemia	1	0	0	1
Hypernatremia	1	0	0	1
Hypertension	0	1	0	1
Hyperuricemia	1	1	0	2
Hypokalemia	2	2	0	4
Hypophosphatemia	1	0	0	1
Hypoxia	0	1	0	1
Hypoxia	0	1	0	1
Ileus	1	0	0	1
Leukocytosis	1	0	0	1
Localized Edema	1	0	0	1
Lymphopenia	1	0	0	1
Nausea	1	0	0	1
Other	11	1	4	16
Pain	1	0	0	1
Penile Infection	1	0	0	1
Platelet Count Decreased	1	2	0	3
Pleural Effusion	1	0	0	1
Pneumonitis	1	0	0	1
Seizure	1	0	0	1
Sepsis	2	1	0	3
Small Intestinal Obstruction	2	0	0	2
Stroke	1	0	0	1
Surgical/Medical Procedures, Other	1	0	0	1
Syncope	0	1	0	1
Tricuspid Valve Disease	1	0	0	1
Tumor Lysis Syndrome	1	0	0	1
Urinary Tract Infection	3	0	0	3
Vomiting	3	0	0	3
**Total**	**94**	**14**	**31**	**139**
**Attribution**				
**Definite**	**12**	**0**	**1**	**13**
**Probable**	**5**	**2**	**0**	**7**
**Possible**	**0**	**0**	**0**	**0**
**Unlikely**	**12**	**3**	**3**	**18**
**Unrelated**	**37**	**6**	**26**	**69**
**Unknown**	**0**	**0**	**0**	**0**
**Missing**	**30**	**2**	**0**	**32**

**Table 5:** Cox Proportional Hazard for Overall and Progression-Free Survival

Value	Coefficient	z	Hazard Ratio	***P***-value
**Overall Survival**				
PNET vs Other	−0.2	−0.5	0.8	0.6
ECOG 1 vs 0	0.1	0.5	1.2	0.7
ECOG 2 vs 0	0.9	2.5	2.7	0.01
Extrahepatic Disease	0.3	0.9	1.3	0.4
Unilobar Treatment	−0.5	−1.9	0.6	0.06
Ascites	1.0	2.0	2.8	0.049
≥ 25% Tumor Burden	−0.3	−1.1	0.7	0.3
**Progression-Free Survival**				
PNET vs Other	−0.2	−0.5	0.9	0.6
ECOG 1 vs 0	−0.09	−0.3	0.9	0.7
ECOG 2 vs 0	0.9	2.5	2.4	0.01
Extrahepatic Disease	0.4	1.4	1.4	0.2
Unilobar Treatment	−0.5	−2.1	0.6	0.03
Ascites	1.6	3.8	4.9	0.0001
≥ 25% Tumor Burden	−0.5	−2.0	0.6	0.049

### PRRT

Nine patients (5%) underwent PRRT. All patients received PRRT after TARE at a median of 21 months after radioembolization (range 12–37 months). In five patients, the indication was extrahepatic progression, while two patients had intra- and extrahepatic progression. Two patients did not have information regarding the indication for PRRT. Two patients developed grade 3 bilirubin toxicities in the setting of progression of hepatic disease 18 and 24 months after PRRT; both patients were 42 months from TARE. No other grade 3 toxicities developed. NET patient enrollment decreased following FDA approval of PRRT in January 2018. From 2015 to 2017, 112 patients were enrolled. From 2018 to 2020, 58 patients were enrolled. This difference was statistically significant (*p* < 0.001).

## Discussion/conclusion

### Discussion

The development of hepatic metastases is a major determinant of OS in patients with metastatic neuroendocrine tumor. The 5-year overall survival of hindgut NET drops from 75 to 88 to 30% and the OS of gastrinoma drops from 95% at 20 years to 15% at 10 years with development of liver metastases [3]. In the current study, we report a median OS of 33 months following resin TARE for NET. Three-year OS was 46%. This group was not treatment naive: 14% of the patients had undergone hepatic resection, 36% had previous arterial interventions, while 34 and 41% had received biologic or cytotoxic chemotherapy. As a result, 36% of the patients were ECOG 1 and 15% were ECOG 2 or greater. Additionally, 45% of the cohort had both bilobar tumor as well as extrahepatic metastases. Patients with ECOG 2 or greater performance status or ascites at time of treatment had shorter OS and PFS. Unilobar therapy and greater than 25% hepatic involvement was associated with longer PFS. In many malignancies, a greater disease burden would predict a shorter PFS. However, with the slow growth rate of many NET, it is possible that the longer PFS with greater than 25% burden was due to the greater tumor volume needed to reach progressive disease. Extrahepatic disease and a pancreatic primary had no statistically significant effect on OS or PFS.

OS in the current study resembles that of the multicenter CIRSE Registry for SIR-Spheres Therapy (CIRT) [15]. OS for the 58 NET patients in that study was 33 months as well. CIRT featured a higher percentage of ECOG 0 patients (65.5% vs 47% in the current study). However, CIRT had more patients with bilobar disease (87.8% vs 45%). Tumor burden was relatively similar between CIRT and the current study (median 20.8% vs 25.7%). OS in the current study is also similar to the 34.4 months reported in 40 patients treated with glass microspheres [21]. Three-year OS in the current study and Memon et al. is also similar: 46% compared with 45% by Memon et al. The ORR and DCR in the current study (44 and 83%, respectively) are also similar to 50 and 86% reported in a TARE metanalysis by Devcic et al. [16]

Subgroup analysis of NET primary locations is inconsistently described in publications using arterial therapy. The current study did not identify a difference in OS or PFS between primary locations. Patients with pancreatic NET are commonly diagnosed at a higher stage than other primaries [23]. This factor likely contributes to the lower OS of this subgroup compared to other subtypes. Many studies group non-pancreatic tumors together as a result. A survival advantage for non-pancreatic NET has been reported following embolization and chemoembolization [24–28]. Gupta et al. reported radiographic response in only 35.2% of pancreatic primaries, compared with 66.7% of carcinoid tumors from other sites [24]. This discrepancy has not been identified with TARE [29, 30]. Devcic et al. described a non-statistically significant trend to longer OS in TARE studies with a lower percentage of pancreatic NET cases, including 70-month OS in a study with 68% small bowel primary tumors [16, 31]. The current study evaluated all subtypes of tumors individually and also compared pancreatic primary tumors to other primary sites grouped together. Pancreatic primary NET had the longest median OS (42 months) of any of the primary sites, although it did not reach statistical significance at Kaplan-Meier or Cox Proportional Hazards analysis. There is no randomized prospective data comparing outcomes of TARE or other arterial therapies. At a minimum, the current study suggests that patients with pancreatic NET can be effectively treated with TARE.

Grade 3 constitutional toxicities were uncommon in the current study, with an incidence of ≤4%. Two recent single center studies focused on longer term toxicities with TARE for NET [17, 18]. Given the potentially long survival for low-grade NET, the North American Neuroendocrine Tumor Society expressed concern expressed about chronic toxicity from TARE when used in routine clinical practice [32]. Tomozawa et al. described new ascites 1 year after treatment in 5/29 (17%) of patients undergoing bilobar therapy [17]. Patients in their study were heavily pretreated with previous resection in 23% and previous embolization in 26%. Baseline ascites was present in 18% of patients, a finding present in 6% of patients in the current study. Tomozawa et al. identified grade 3 hepatic function toxicities in 4 of 52 patients (8%) 1 year after treatment [17]. By contrast, the current study identified new ascites in 8 (5%) patients without progressive disease at any point of follow-up and long-term grade 3 hyperbilirubinemia in 5 (3%) patients. The current findings more closely mirror those described by Su et al. [18] While they identified 39 patients out of 54 treated who had new ascites or thrombocytopenia, confounding variables such as hepatic progression were present in 37 patients, leaving 2 (5%) with toxicity that was clearly attributable to TARE. Currie et al. reviewed incidence of chronic hepatitis following treatment of 91 patients: 63 and 28 underwent chemoembolization and TARE, respectively. The incidence of grade 3 toxicities attributable to treatment with TARE (14%) and chemoembolization (3%) did not reach statistical significance, potentially due to sample size [33].

Only one of the 15 patients (6.7%) with colonized bile ducts developed a hepatic abscess following TARE. Devulapalli et al. reported a 7.9% incidence of abscesses in a multi-center review of 126 patients, including 40 with NET, where antibiotic prophylaxis was used in 151 (83.8%) TARE procedures. Infectious complications were identified in ten patients after 11 procedures. Nine patients in the current group underwent subsequent PRRT with two grade 3 bilirubin toxicities developing in patients with intrahepatic progression at end of life. Enrollment of NET patients in the current study decreased after publication of the NETTER-1 data in 2017 with subsequent FDA approval: 112 NET patients were enrolled from 2015 to 2017 and 58 from 2018 to 2020 [22, 34]. There is limited data of patients who have undergone TARE and PRRT [32].

The current study contains limitations. Sites entered data at self-regulated time points, resulting in less than 100% entry and evaluation at non-uniform time points. The sample size of this study is relatively small. However, it is the largest multicenter report of TARE in NET patients to date. Dosimetry was largely performed using body-surface area methodology, which has been standard practice until 2021 [35]. Finally, in assessing long-term toxicity, imaging findings such as splenic and liver volumetrics were not available, although other factors such as ascites and hepatic function toxicities were tracked.

### Conclusion

RESiN demonstrates effective therapy for patients with NELM with median OS of 33 months and PFS of 25 months. In a population, toxicity profiles were favorable. Overall, there is no significant OS or PFS difference among different NET primary locations, though we observed the longest OS in NELM patients with pancreatic primary.

## Data Availability

All data generated or analyzed during this study are included in this article. Further enquiries can be directed to the corresponding author.
